# Amino acids predict prognosis in patients with acute dyspnea

**DOI:** 10.1186/s12873-021-00519-y

**Published:** 2021-10-30

**Authors:** Karolin Wiklund, Klas Gränsbo, Peter Almgren, Marjaneh Peyman, Lena Tegnér, Maria Toni-Bengtsson, Mattias Wieloch, Olle Melander

**Affiliations:** 1 The Department of Clinical Sciences Malmo, Faculty of Medicine, Lund University, Malmo, Sweden; 2 The Department of Anesthesiology and Intensive care, NU-Hospital group, Trollhattan/Uddevalla, Sweden; 3 The Department of Internal Medicine and Emergency Medicine, Skane University Hospital, Malmo, Sweden

**Keywords:** Dyspnea, Emergency service, hospital, Glycine, Phenylalanine, Valine, Amino acids

## Abstract

**Background:**

To identify amino acids that can predict risk of 90-day mortality in patients with acute dyspnea.

**Method:**

Plasma levels of nine amino acids were analyzed 663 adult patients admitted to the Emergency Department (ED) with acute dyspnea. Cox proportional hazards models were used to examine the relation between amino acid levels and the risk of 90-day mortality.

**Result:**

Eighty patients (12.1%) died within 90 days of admission. An “Amino Acid Mortality Risk Score” (AMRS), summing absolute plasma levels of glycine, phenylalanine and valine, demonstrated that among the patients belonging to quartile 1 (Q1) of the AMRS, only 4 patients died, compared to 44 patients in quartile 4. Using Q1 of the AMRS as reference, each increment of 1 SD in the AMRS was associated with a hazard ratio (HR) of 2.15 for 90-day mortality, and the HR was > 9 times higher in Q4.

**Conclusion:**

Glycine, phenylalanine and valine are associated with a risk of 90-day mortality in patients admitted to the ED for acute dyspnea, suggesting that these amino acids may be useful in risk assessments.

## Background

One of the most common symptoms in the emergency departments (ED) is dyspnea, presented in roughly 3–4 million annual ER visits in the United States [1] In addition to identifying the correct diagnosis and therapy, ED physicians have to make decisions regarding monitoring, level of care, or the time to follow-up in the case of hospital discharge. One of the key factors to consider when making these decisions is the patient’s risk of long-term mortality.

Chronic cardiorespiratory diseases, which underlie susceptibility to episodes of acute dyspnea, and acute events leading to an exacerbation, pose severe challenges to the body and result in metabolic changes, [2, 3] often mediated by the release of stress hormones. This leads to a switch from an anabolic to a catabolic state with acute insulin resistance, sometimes unmasked clinically as temporary hyperglycemia, and muscle breakdown [4]. Interestingly, in the non-acute setting, elevated plasma concentrations of the branched-chain and aromatic amino acids phenylalanine, isoleucine and tyrosine, are related to insulin resistance and predict the development of both diabetes mellitus and cardiovascular disease [5, 6]. After a standardized protein meal, healthy subjects with relatively higher degree of insulin resistance and glycemia show higher post meal responses of phenylalanine, isoleucine and tyrosine, suggesting that insulin resistance is linked to defective insulin-mediated uptake of these amino acids [7]. The hypothesis in this study is that insulin resistance, induced by stress in patients with acute dyspnea, would alter levels of some of these amino acids, reflecting the severity of the acute disease state. Additionally, as the stress hormone-induced catabolic state in acute dyspnea provides energy substrates to vital organs, the assumption is that the severity of the disease state in patients with acute dyspnea may also be related to increased levels of specific amino acids. Increased levels of these amino acids may reflect muscle breakdown, whereas reduced levels of other circulating amino acids could rather be a consequence of increased substrate consumption. Hence, the aim of the current study was to investigate if circulating plasma levels of specific amino acids, selected based on prior literature implicating their role in metabolic diseases, would reflect the severity of acute disease state, predicting short-term mortality among patients admitted to the ED with acute dyspnea.

## Methods

### Study population

This study was conducted at Skane University Hospital (SUS Malmo), which has almost 85,000 ED visits per year and a catchment area of around 400,000 residents, as part of a larger research project. In summary, the data collection follows the format published in Wiklund et al. (2016) [8] in which adult patients presenting in the ED with a primary symptom of acute dyspnea were included in the study between March 62,013, and July 292,015. Patients were only included during office hours, i.e., 06:45 AM-4:30 PM, when a research nurse was present. The participants were assigned a triage category of 1–4 using the “Medical Emergency Triage and Treatment System-Adult” score (METTS-A) and underwent routine blood chemistry examinations The METTS-A is a five-level triage-tool and was the standard of triage for patients during the time of inclusion [9].. The tool evaluates vital signs (oxygen saturation, respiratory rate, heart rate, blood pressure, level of consciousness and body temperature), as well as symptoms. Patients are triaged into five levels: (i) red, priority 1 – indicating a life threatening state; (ii) orange, priority 2 – signaling a potentially life threatening state; (iii) yellow, priority 3 – indicating a patient in need of medical attention within 2 h but is not in a life threatening state; (vi) green, priority 4 - not in need of immediate emergency care; and (v) blue, priority 0 – indicating a patient who could be referred to primary care.

This study included 784 patients, of which 121 were later omitted due to missing values in the variables of interest, with a final sample size of 663 patients.

### Clinical parameters

Blood pressure, oxygen saturation and heart rate were measured using a fully automated oscillometric device (CARESCAPE Monitor B850 or B650, General Electric Healthcare) [10, 11]. Level of consciousness was determined using the “Reaction Level Scale” (RLS) [12]. Respiratory rate was manually counted by an ED nurse. After study inclusion, patients were interviewed regarding their smoking status (never smoked/former smoker/active smoker, which included occasional smoker), prevalence of chronic diseases known to cause dyspnea (including congestive heart failure (CHF), chronic obstructive pulmonary disease (COPD), asthma, coronary artery disease (CAD), atrial fibrillation, restrictive lung disease, cancer, thromboembolic disease, and rheumatic disease), and current medications. The research nurses also examined the patient’s medical records validating any previously reported diagnoses.

The primary endpoint was all-cause mortality within 90 days from presentation to the ED. The patients’ personal identification numbers, linked to Swedish the national civil registry, were used to retrospectively collect survival status up to 90 days after presentation, as well as date of death, if the patient was deceased.

### Assays

Plasma concentration of CRP, lactate and creatinine was measured using a Radiometer ABL800 Flex machine [13] or Afinion AS100 Analyzer System [14]. Estimated glomerular filtration was calculated according to the Lund – Malmo creatinine-based glomerular filtration rate prediction eq. (LM) instead of a globally established model (MDRD) since the LM equation was primarily validated using creatinine assays for analysis in the present laboratory. In the present population, the LM equation performs better than the MDRD equation, with most apparent benefits in patients with eGFR< 30 and 30–59 ml/min/1.73 m2 [15]. Blood samples were collected immediately at presentation in the ED, centrifuges with separation of serum and plasma, and were stored at − 80 °C for later analysis of levels of amino acids.

The frozen serum samples were analyzed using a proton nuclear magnetic resonance (NMR) platform (Bruker AVANCE III, 500 MHz spectrometer), which includes the nine selected amino acids chosen for revision in the current study (Table 2) [16].

### Statistical analysis

Due to the skewed distribution nature of the data, natural logarithms were derived for the values of amino acids, CRP and lactate, achieving statistical normality. The log-transformed values were expressed using a standardized scale (per 1 standard deviation increment). In the Cox proportional hazard models, the dependent variable was 90-day mortality and the independent variables consisted of the levels of each amino acid respectively. The follow-up time started at the time of presentation to the ED and lasted to the time of death (if death occurred) or to the end of the study, i.e., 90 days after presentation (if death did not occur). The first cox proportional hazard model (model 1) was adjusted for sex and age. The amino acids with a significant association to 90-day mortality (*p*-value < 0.05) were entered into model 2, including adjustments for sex, age, METTS-A score, oxygen saturation, respiratory rate, CRP, eGFR and lactate. Amino acids that demonstrated a significant relationship with 90-day mortality in model 2 were summed up to a score, the “Amino acid Mortality Risk Score” (AMRS) and weighted using their beta-coefficients from the Cox proportional hazards model (i.e., β-coefficient for amino acid 1 x Z-score of amino acid 1 + β-coefficient of amino acid 2 x Z-score of amino acid 2). The sum of the weighted values of amino acids was expressed as multiples of 1 SD (z-score), which were then ranked and ordered into quartiles.

Finally, the improvement of discrimination was assessed using Harrell’s c-index and risk reclassification when adding information obtained from amino acids on top of traditional risk stratification parameters (sex, age, METTS-A score, oxygen saturation, respiratory rate, CRP, eGFR and lactate) using categorized Net Reclassification Improvement (NRI) [17–19]. A *p*-value of < 0.05 was considered significant. Additional adjustments were performed for comorbidities and smoking status (current, former and never smoker). Data were analyzed using IBM SPSS statistics 22 (SPSS) (SPSS Inc., Chicago, IL, USA) and Stata version 8 (Stata Corp, College Station, Texas).

The study was approved by the ethics board at Lund University, Sweden and followed the precepts established by the Declaration of Helsinki.

## Results

Out of a total study population of 663 patients (Table 1), 404(61%) were admitted to a ward, of which 137 were admitted to the Intensive Care Unit, Cardiac Care Unit or Acute Medical Ward (e.g., a ward with continuous monitoring capabilities). During the 90 days of follow-up, 80 patients died, 69 of whom had been admitted to a ward upon their arrival in the ED, and 6 patients died in the ED.

**Table 1:** Clinical characteristics of study population (*n* = 663)

Age years mean ± SD	71.54 ± 17.3
Sex Female (%)	354 (53.4)
METTS-A^a^ (%)	45 (6.8) / 305 (46.0) / 238 (35.9) / 75 (11.3)
Oxygen saturation % mean ± SD	93.4 ± 6.4
Respiratory Rate mean ± SD	24.3 ± 6.9
CRP mg/l median (IQR)	10 (3.6–36)
Lactate mmol/l median (IQR)	1.5 (1.2–2.1)
eGFR ml/min median (IQR)	62.1 (43–78.8) 4 unknown
CHF (%)	231 (34.8) 3 unknown
COPD (%)	203 (30.6) 4 unknown
Smoking^b^ (%)	193 (29.1) / 338 (51.0) / 115 (17.3) 15 unknown

^a^4 categories of METTS-A priority 4/3/2/1 least critical to most critical

^b^3 categories non-smokers/former smokers/active smokers

Levels of three of the amino acids in model 1, valine, phenylalanine and glycine, demonstrated a significant association with 90-day mortality (Table 2). In model 2 (HR and 95% CI expressed per 1 standard deviation increment of the respective amino acid concentration), each of these three amino acids remained significantly and independently related to 90-day mortality: phenylalanine (HR = 1.60 (CI 1.18–2.19) *p* = 0.0028) valine (HR = 0.48 (CI 0.37–0.62) *p* = 2.91 × 10^− 8^) and glycine (HR = 1.51 (CI 1.21–1.88) *p* = 0.00026). In addition, age (per year) (HR = 1.06 (CI 1.03–1.08) *P* = 0.000001) and lactate (mmol/l) (HR = 1.25 (CI 1.01–1.55) *P* = 0.039) significantly and independently predicted 90-day mortality.

**Table 2:** Relationships between plasma levels of amino acids and risk of 90 day-mortality

Amino acid	Hazard Ratio adjusted for Age and Sex (95% CI)	*P*-value
Alanine	0.91 (0.73–1.13)	0.41
Glutamine	0.96 (0.77–1.21)	0.75
Glycine	1.32 (1.08–1.62)	7.2 × 10^−3^
Histidine	0.83 (0.65–1,06)	0.13
Isoleucine	0,86 (0.68–1.08)	0.18
Leucine	0.79 (0.63–1.01)	0.06
Phenylalanine	1.53 (1.24–1.88)	7.2 × 10^− 5^
Tyrosine	1.08 (0.87–1.35)	0.49
Valine	0.61 (0.49–0.77)	2.2 × 10^− 5^

**Fig. 1:**
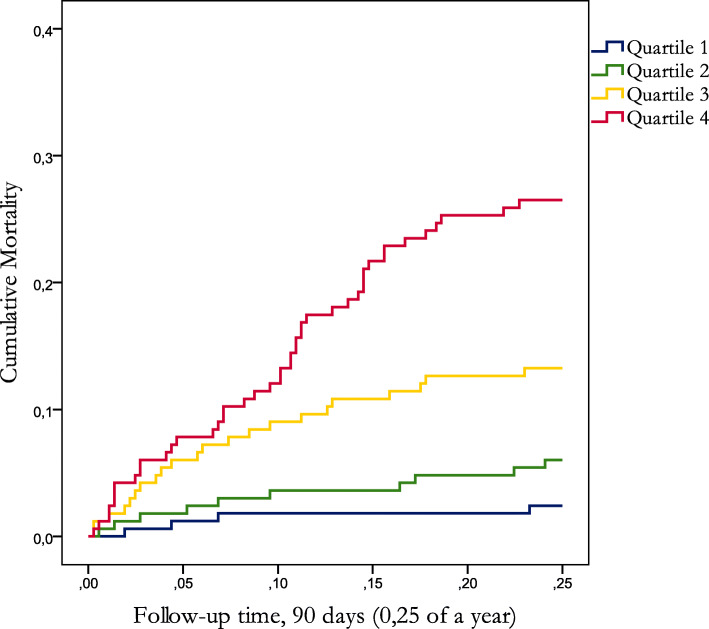
Kaplan-Meier plot showing cumulative mortality during 90 days of follow-up. Quartile 1 denotes the lowest values of the Amino Acid Risk Score (AMRS) and Quartile 4 the highest values

The weighted and summed AMRS of glycine, phenylalanine and valine was strongly associated with risk of 90-day mortality (Table 3), and quartile analyses demonstrated that mortality was low in subjects with values below the median of the summed biomarker score and increased steeply after quartile 2–3(Fig. 1). In quartile 1 the 90-day mortality was only 2.4%, whereas it increased to 26.5% in quartile 4.

**Table 3:** Multivariate adjusted hazard ratios for the Amino acid Mortality Risk Score (AMRS) in relation to 90-day mortality

	All patients	Quartile 1	Quartile 2	Quartile 3	Quartile 4
N/N events	663/80	165/4	166/10	166/22	166/44
HR (95% CI)a	2.15 (1.65–2.82)	1	1.97 (0.61–6.36)	4.12 (1.40–12.18)	9.33 (3.24–26.90)

HR: *P*-value: 2,2 × 10^− 8^, P-trend 4,9 × 10^− 7^.

^a^Adjusted for age, sex, METTS-A, oxygen saturation, respiratory rate, CRP, lactate and eGFR

The ability of the amino acid score to discriminate between 90-day survival and death was assessed using Harrell’s c-index. In a model with only traditional risk factors, the c-index (risk reclassification between 90-day survival and non-survival) was 0.76 (95% CI 0.72–0.81), which increased by more than 5 percentage points to 0.819 (95% CI 0.78–0.86) when the AMRS was added on top of the traditional risk stratification parameters (sex, age, METTS-A score, oxygen saturation, respiratory rate, CRP, e-GFR and lactate).

Using the net reclassification improvement (NRI) [17] at a cutoff “risk level” of 1% for 90-day mortality, the AMRS correctly re-classified 6.9% of the patients who did not die during the follow-up from above to below 1% of the estimated 90-day mortality risk when added on top of traditional risk stratification parameters. The AMRS incorrectly re-classified 1.7% of the surviving patients from below to above 1% risk. As none of the patients who died during follow-up had an estimated 90-day mortality risk below 1%, the overall NRI was 5.1% (*P* = 0.000022).

Smoking status, history of CHF and history of COPD were separately added to model 2 adjustments; none of these factors were significant, and their presence in the model did not change the relationship between the AMRS and 90-day mortality (data not shown).

## Discussion

Plasma concentration of three amino acids, i.e., high levels of phenylalanine and glycine and low levels of valine, measured upon presentation at the ED, were shown in the study to predict mortality strongly and independently in an unselected cohort of patients with acute dyspnea, irrespective of underlying pathology.

Measurable changes in metabolism, especially those pertaining to when the body enters a catabolic state, as demonstrated via pathophysiology, can indicate both severity of a disease and its prognosis. This has been previously demonstrated in several studies, including those involving heart failure, COPD, and septicemia [20–22]. During a metabolic shift to a catabolic state, stress hormones are released leading to an increase in proteolysis, decreased lipolysis and an increased insulin resistance [23]. Seeing as CHF, COPD, and sepsis (pneumonia) are some of the more severe conditions associated with patients presenting with acute dyspnea, it would seem logical that changes levels of amino acids could in fact indicate a higher mortality risk.

In population-based studies, phenylalanine has been shown to be related to insulin resistance and to strongly predict cardiovascular disease [24, 25]. Acute dyspnea, in its catabolic state, involves increased skeletal muscle breakdown which in turn leads to a release of certain amino acids from these muscles. The hypothesis is that the hepatic insulin mediated uptake and metabolism of phenylalanine is reduced due to an acute insulin resistance and inflammation, resulting in high levels of phenylalanine in the patients with a more severe disease. Valine, on the other hand is a branched chain amino acid, like isoleucine and leucine and will be metabolized and used in gluconeogenesis to a higher/greater extent due to its properties in a catabolic state. When peripheral tissues lack energy substrate, branched chain amino acids are the preferred energy substrate over non-branched amino acids [20, 21, 23, 26]. All branched-chain amino acids were generally decreased in non-survivors in this study, although only valine was significantly decreased, which could explain why valine is inversely correlated and phenylalanine is directly correlated to an increased 90-day mortality. In addition to phenylalanine, levels of glycine significantly predicted 90-day mortality, and in experimental studies, glycine has been suggested to protect against cell death and ischemia reperfusion injury. Glycine has also been tested with some positive results as an anti-inflammatory therapy in patients with cystic fibrosis [27–29]. Given the results of these previous studies, it is likely that the higher levels of glycine, which correlated to poorer outcomes in this analysis, could be an endogenous compensatory mechanism to overcome circulatory and respiratory stress. However, the mechanisms underlying the associations between plasma levels of phenylalanine, valine and glycine and excess 90-day mortality need to be elucidated in other studies, since this was not a mechanistic study.

The findings in this analysis have several clinical implications, especially in the acute setting of the ED. Although there are multiple tools for assessing risk and severity at the ED such as early lab works and vital signs, AMRS performed better and was statistically independent of several parameters in this study, such as initial vital signs, CRP, comorbidities, eGFR and the triage algorithm score (METTS-A), the latter of which has previously been demonstrated to predict in-hospital mortality [9].

There was a large difference between 90-day mortality between patients in the bottom and top quartile of the AMRS. The mortality rate of 12% in the total patient population in this study was notably high, and with the relatively short-term follow-up time, one could argue that the mortality rate reflects the severity of disease state at the time of presentation to the ED. In summary the AMRS may be of added value for a first-line physician, predicting a safe discharge of the patient with or without follow-up in primary care. The AMRS may also be used to support early prioritization of intensive care and monitoring since almost one-third of patients in the top quartile of the AMRS died within 90 days and this could lead to a speculation that these patients may have benefited from a more intensive care approach. Given the high 90-day mortality in the patient group with high AMRS, there may also be a need for a closer follow-up, or an early visit at a hospital outpatient clinic if the patient isn’t eligible for admission upon presentation to the ED.

As previously stated, there are many conditions with a variety of treatment regimens that presents with acute dyspnea and this study has demonstrated that AMRS could support prioritizing and triage of patients under this category. A patient with high AMRS may be prioritized towards more extensive medical testing and examinations in-hospital, it may lead to quicker diagnosis and its root cause, as well as possibly staging and initiating short-term and long-term treatments. On the other hand, for a patient with a low AMRS the sense of urgency is lower and the need for further testing and examinations could be evaluated at a later point outside of the ED, possibly on primary care level.

A model using AMRS in patients with acute dyspnea in the ED, could also assist in creating a more medically justified distribution of the time and budget of the ED as well as determine resources for adequate follow-up. Future research is however needed to specifically address these aspects.

### Limitations

Although there is not an established ideal time for follow-up of acute dyspnea, a 90 day follow- up is regularly applied in ED-based biomarker studies. An alternative endpoint could have been in-hospital mortality, but this measurement would have provided issues with standardization and was therefore dismissed. Thirty-day mortality is another alternative endpoint that is likely to have a relation to the acute underlying disease causing dyspnea, along with in-hospital mortality. While a shorter follow-up time than 90 days, may lead to an underestimation of fatality cases which were related to the initial symptom of dyspnea upon presentation to the ED, the Kaplan-Meier plot (Fig. 1) has an almost linear growth over 90 days, implying that the hazard ratio would have been just as high using a 30-day endpoint.

A relatively long follow-up time would however be particularly important when considering the potential clinical implication of using the AMRS to identify patients with low values and low risk and to use this as a clinical support to safely discharge them, with little or no need for follow-up. In the end, ninety days of follow-up is a reasonable compromise to include prioritizing higher level of care, need for follow-up, but in particular to support a safe discharge of the patient with acute dyspnea.

## Conclusion

Glycine, phenylalanine and valine are associated with the risk of 90-day mortality in patients admitted to the ED with a primary symptom of acute dyspnea. A score using these amino acids may guide in risk assessment, supporting decision making processes to establish appropriate level of care and treatment intensity.

## Data Availability

The data that support the findings of this study are available on request from the corresponding author K.W. The data are not publicly available due to them containing information that could compromise research participant privacy/consent.

## Abbreviations

AMRS: Amino acid mortality score
ED: Emergency department
eGFR: Estimated glomerular filtration rate
CAD: Coronary artery disease
CHF: Congestive heart failure
CI: Confidence interval
COPD: Chronic obstructive pulmonary disease
HR: Hazard ratio
LM: Lund – Malmo creatinine-based glomerular filtration rate prediction equation
MDRD: Modification of Diet in Renal Disease
METTS-A: Medical Emergency Triage and Treatment System-Adult
NMR: Proton nuclear magnetic resonance
NRI: Net reclassification index
Q: Quartile
RLS: Reaction level scale
SD: Standard deviation
SUS Malmo: Skane University Hospital Malmo
